# When does exercise hinder rather than halt tumor progression? The decisive role of immunotherapy responsiveness

**DOI:** 10.1073/pnas.2616250123

**Published:** 2026-07-17

**Authors:** Xin Zhou, Jingjing Liu, Qinglu Wang

**Affiliations:** ^a^ Graduate School of Education, Shandong Sport University, Jinan 250102, China

A recent study published in *PNAS* provided the first systematic evidence that, in a 4T1 triple-negative breast cancer mouse model, mild caloric restriction combined with chemotherapy reduced primary tumor growth and pulmonary metastasis; however, the addition of voluntary exercise did not further suppress primary tumor growth and instead attenuated the protective effect of caloric restriction against lung metastasis (1). This finding challenges the prevailing assumption that exercise is invariably beneficial in cancer treatment and suggests that the antitumor value of exercise interventions is highly context-dependent rather than universally applicable across all tumor types and therapeutic settings.

One possible explanation for this phenomenon lies in the dynamic nature of the tumor microenvironment. Although exercise has demonstrated antitumor potential in certain malignancies, such as pancreatic cancer and non–small cell lung cancer, through mechanisms including improved systemic metabolic status, enhanced cardiopulmonary function, and increased immune cell infiltration, the tumor microenvironment itself is continuously shaped by abnormal angiogenesis, immunosuppression, and metabolic reprogramming (2, 3). These ongoing changes make the beneficial effects of exercise on tumor control far from straightforward. In clinical practice, tumors are often broadly categorized as “hot” or “cold” according to the degree of immune cell infiltration and immune activation, with “hot tumors” characterized by abundant immune infiltration and relatively active antitumor responses, whereas “cold tumors” exhibit limited immune cell presence or functionally constrained infiltration (4). However, we propose that this classification based primarily on static immune status is insufficient to fully capture the vascular, metabolic, and microenvironmental remodeling induced by exercise. Since the anticancer effects of exercise are not determined by a single metabolic state, the suitability of a tumor for exercise intervention should not be inferred solely from its immune phenotype.

On this basis, we hypothesize that the efficacy of exercise as a tumor intervention may be closely associated with the tumor’s responsiveness to immunotherapy; indeed, sensitivity to immunotherapy may be an important prerequisite for exercise to exert meaningful antitumor effects. Previous studies have shown that exercise can enhance the efficacy of immune checkpoint inhibitors and suppress tumor growth by increasing microbiota-derived formate, thereby strengthening CD8^+^ T cell function (5). In melanoma-bearing mice treated with anti-programmed cell death protein 1 (PD-1) antibodies, exercise likewise significantly reduced tumor weight and volume, producing a stronger inhibitory effect on tumor growth (6). Collectively, these findings indicate that whether exercise can truly serve as an effective adjunctive antitumor strategy may depend largely on whether the tumor possesses sufficient immune plasticity and an adequate foundation for immune responsiveness.

Therefore, exercise intervention should not be regarded as a universally applicable “anticancer strategy,” but rather should be reevaluated within a more precise stratification framework. Only when the specific biological background and therapeutic context are clearly defined can exercise be meaningfully incorporated into cancer intervention as an effective component, rather than being broadly promoted in a generalized manner.

## Data Availability

There are no data underlying this work. Other data are included in the article.
